# Effects of melatonin on *in vitro* oocyte maturation and embryo development in pigs

**DOI:** 10.14202/vetworld.2025.1234-1241

**Published:** 2025-05-21

**Authors:** Laura Andrea Blancas-Alvarez, Alma Lilia Alvarez-Guerrero, Alicia Alcantar-Rodriguez, Alfredo Medrano

**Affiliations:** 1 Laboratory of Animal Reproduction (L2), Multidisciplinary Research Unit (UIM), Faculty of Superior Studies, Cuautitlan, National Autonomous University of Mexico, Cuautitlan Izcalli 54714, Mexico; 2 Department of Comparative Biology, Faculty of Sciences, National Autonomous University of Mexico, CDMX 04510 Mexico

**Keywords:** cumulus-oocyte-complexes, embryo development, *in vitro* maturation, melatonin, pigs

## Abstract

**Background and Aim:**

*In vitro* fertilization (IVF) efficiency in pigs remains suboptimal, partly due to oxidative stress during oocyte maturation and embryo development. Melatonin (MLT), an endogenous antioxidant, has been proposed as a beneficial supplement in reproductive culture systems. This study aimed to evaluate the effects of different concentrations of MLT on *in vitro* porcine oocyte maturation and subsequent embryo development.

**Materials and Methods:**

Ovaries were obtained from prepubertal gilts at a local slaughterhouse. A total of 1142 cumulus-oocyte-complexes (COC) were allocated into four groups and matured *in vitro* with 0, 1, 3, or 5 μM MLT for 44 h. Oocyte maturation was assessed using aceto-orcein staining and viability with trypan blue staining. Subsequently, 1312 COC underwent IVF using a standardized sperm concentration, followed by embryo culture in North Carolina State University-23 medium supplemented with corresponding MLT concentrations for 8 days. Embryo development was classified according to cleavage, morula, and early blastocyst stages. Data were analyzed using analysis of variance with Tukey’s *post hoc* test (p < 0.05).

**Results:**

Although supplementation with 1.0 μM MLT resulted in the highest metaphase II oocyte maturation rate (45.9% ± 5.70%) and blastocyst formation (26.9% ± 9.57%), no significant differences were observed among treatments in either oocyte maturation, COC viability, or embryo development stages (p > 0.05).

**Conclusion:**

Supplementation with 0–5 μM MLT during oocyte maturation and embryo culture did not significantly enhance *in vitro* maturation rates or embryonic developmental outcomes in pigs. Further investigations are warranted to optimize MLT concentrations and elucidate its mechanistic role during porcine oocyte and embryo *in vitro* culture.

## INTRODUCTION

Pigs are highly significant in both agricultural production and biomedical research, serving as major livestock species and valuable animal models for human biomedical studies due to their close physiological similarities to humans. Numerous technological advancements in reproductive biotechnology have been established, encompassing a wide range of techniques such as artificial insemination, embryo transfer, gamete and embryo cryopreservation, sperm sexing, *in vitro* embryo production, nuclear transfer, and DNA construct microinjection [1]. Despite the extensive use of *in vitro* fertilization (IVF) as a reproductive biotechnology tool, fertility rates achieved through this technique remain suboptimal. Several factors, including culture media composition, metal exposure, pH fluctuations, atmospheric oxygen tension, and oxidative stress induced by visible light, contribute to frequent abnormalities during *in vitro* embryonic development [2].

Melatonin (MLT) (N-acetyl-5-methoxytryptamine) is a lipid-soluble endogenous neurohormone predominantly synthesized by the pineal gland. It plays a pivotal role in regulating both seasonal and circadian rhythms [3]. In addition to its regulatory functions, MLT is widely recognized as a potent antioxidant. Its mechanisms of action include direct scavenging of free radicals, induction of antioxidant enzymes such as glutathione reductase and glutathione peroxidase, and inhibition of peroxidase nitric oxide synthase activity [4].

In women and sows, MLT is produced in the ovary, where it is involved in both follicular and oocyte maturation processes [5, 6]. Furthermore, MLT confers protection against oxidative damage in spermatozoa, thereby preserving their viability [7]. These protective roles are mediated through both receptor-dependent and receptor-independent mechanisms [8, 9]. Although MLT has been demonstrated to promote embryonic development across multiple species, including pigs [6], the optimal concentration for *in vitro* supplementation remains inconsistent and inconclusive among different studies.

Although MLT has been extensively studied for its antioxidant properties and its ability to enhance oocyte quality and embryonic development in various species, findings regarding its optimal concentration during *in vitro* maturation and embryo culture remain inconsistent and inconclusive. Prior studies in pigs have reported conflicting outcomes, likely due to differences in experimental designs, MLT dosages, and culture conditions. Moreover, while MLT’s protective effects against oxidative stress are well-documented, its specific impact on porcine cumulus-oocyte-complexes (COC) and subsequent embryo development under standardized *in vitro* conditions remains poorly characterized.

Therefore, the present study aimed to evaluate the effects of different concentrations of MLT (0, 1, 3, and 5 µM) during *in vitro* maturation of porcine oocytes and subsequent embryo development. By standardizing experimental conditions, this study seeks to clarify whether MLT supplementation enhances oocyte nuclear maturation, viability, and embryonic developmental competence, thereby contributing to a more defined understanding of MLT’s role in porcine reproductive biotechnology.

## MATERIALS AND METHODS

### Ethical approval

This study did not require approval from the Subcommittee for the Care of Animals in Experimentation at the Facultad de Estudios Superiores Cuautitlán, National Autonomous University of Mexico, as it exclusively involved post-mortem collection of ovaries and oocytes from an authorized slaughterhouse, without direct experimentation on live animals.

### Study period and location

This study was conducted from August 2022 to November 2023 at the Laboratory of Reproduction (L2), Multidisciplinary Research Unit, Facultad de Estudios Superiores Cuautitlán, National Autonomous University of Mexico.

### Materials

The Tyrode’s Lactate-4-(2-Hydroxyethyl) piperazine-1-ethanesulfonic acid, N-(2-Hydroxyethyl) piperazine-N’-(2-ethanesulfonic acid)-Poly(vinyl alcohol) (TL-HEPES-PVA) solution, Tissue culture medium-199 (TCM-199) containing bicarbonate and Earle’s salts, fertilization medium tris buffer modified (mTBM), and North Carolina State University-23 (NCSU-23) embryo development medium (USA) were obtained from In vitro Mexico. Fatty acid-free bovine serum albumin (BSA) (BSA, Fraction V) and mineral oil were obtained from Sigma-Aldrich, Mexico. Glacial acetic acid-ethanol solution (1:3 v/v) was obtained from JT Baker, Mexico.

Petri dishes were obtained from Falcon (USA) and BD (Mexico); conical tubes were obtained from SPL-Life Sciences (Korea); hypodermic syringes without rubber plungers and 18-gauge needles were obtained from Norm-Ject (USA) and SensiMedical (Mexico), respectively.

The Vortex shaker was obtained from Scientific Industries (USA); the Neubauer chamber was sourced from Hausser Scientific (Germany); and the automatic incubator (MCO18AC) was from SANYO (Japan). Stereoscopic (S7E), optical (DM1000), and inverted (DMIL LED) microscopes were obtained from Leica (Germany); the USB Digital Camera (OPT-14mp) was obtained from Optisum (China).

### Experimental design

This work was conducted in two stages. The first stage involved *in vitro* oocyte maturation, in which four concentrations – 0.0, 1.0, 3.0, and 5.0 μM MLT – were tested; a total of 1142 COC were incubated for 44 h. This experiment was replicated six times.

The second stage was split into two parts: (i) IVF, during which matured oocytes and spermatozoa were co-incubated for 7 h, and the fertilization medium was not supplemented with MLT and (ii) *in vitro* embryo development, during which, after IVF, probable zygotes (n = 1312) were again supplemented with 0.0, 1.0, 3.0, and 5.0 μM MLT and incubated for 7 days. This experiment was also replicated 6 times.

### *In vitro* oocyte maturation

#### Collection of ovaries and COC

Ovaries were collected from prepubertal gilts slaughtered at an authorized abattoir and transported to the laboratory in a physiological saline solution (0.9% NaCl, w/v) maintained at 38°C within 2 h of collection. On arrival at the laboratory, the ovaries were washed twice with the same solution at 38°C.

Follicular fluid was aspirated from the antral follicles (3–6 mm in diameter) using a 10 mL hypodermic syringe without a rubber plunger (Norm-Ject, USA) and an 18-gauge needle (SensiMedical, Mexico).

Follicular fluid was deposited in 50 mL conical tubes and allowed to settle for 20 min to obtain the cellular pellet. Then, the supernatant was removed, and the cellular pellet was washed twice with 2.0 mL of TL-HEPES-PVA solution (In vitro).

The suspension was then left standing for 15 min in an automatic incubator (SANYO MCO18AC, Japan) at 38.5°C with 5% CO^2^ and a saturated humidity atmosphere. Subsequently, the cellular pellet was placed in a Petri dish (100 × 100 mm, Falcon) and observed under a stereoscopic microscope with a 7× objective (Leica) to localize the COC, which were morphologically assessed.

COCs were selected according to the classification by De Loos *et al*. [10], and only those of quality grades 1 and 2 (intact zona pellucida, homogeneous appearance of oocyte cytoplasm, and at least 2–6 layers of cumulus cells surrounding the oocytes) were used in these experiments.

#### In vitro maturation

The selected COC was washed three times in 500 μL drops of TCM-199 medium containing bi- carbonate and Earle’s salts (In vitro).

Four MLT concentrations – (i) 0.0, (ii) 1.0, (iii) 3.0, and (iv) 5.0 μM – were tested during *in vitro* oocyte maturation as follows: Control Group (CG) with 500 μL of TCM-199 medium without MLT; Experimental Group 1 (EG1) with 500 μL of TCM-199 medium supplemented with 1.0 μM MLT; Experimental Group 2 (EG2) with 500 μL of TCM-199 medium supplemented with 3.0 μM MLT; and Experimental Group 3 (EG3) with 500 μL of TCM-199 medium supplemented with 5.0 μM MLT.

Approximately 30 COC per treatment group were transferred to four-well culture dishes, overlaid with 200 μL of mineral oil (Sigma-Aldrich), and incubated at 38.5°C under 5% CO^2^ and saturated humidity for 44 h.

#### Assessment of in vitro maturation

Following *in vitro* maturation, COC were denuded using a vortex shaker (Scientific Industries, USA). Each treatment group’s COC was placed in 15 mL conical tubes (SPL-Life Sciences, Korea) containing 1.0 mL of TL-HEPES-PVA medium at room temperature (~23°C).

These tubes were placed in the vortex shaker for 90 s at maximum speed. Subsequently, denuded COC were placed in 5 × 5 cm Petri dishes (BD). Samples were observed under a stereoscopic microscope (Leica) to recover the COC.

To assess maturation, 20 oocytes from each treatment were stained with 1% aceto-orcein: Oocytes were placed on a slide, pressed using a coverslip fixed with a paraffin–petrolatum (1:1) mixture, and fixed in a glacial acetic acid-ethanol solution (1:3 v/v) for 48 h.

Oocytes were stained with 1% orcein solution in 45% acetic acid; excess stain was removed by washing in a solution of water, glacial acetic acid, and ethanol (3:1:1 v/v/v) for 3 min.

Slides were evaluated under an optical microscope (Leica) at 40× magnification.

Oocytes were classified into four categories based on nuclear and cytoplasmic morphology: (i) Immature (germinal vesicle [GV] stage) with homogeneous cytoplasm and a large, central or slightly displaced nucleus; (ii) maturing (Metaphase I [MI] stage) with nucleus displaced towards the periphery without contacting the plasma membrane; (iii) mature (Metaphase II [MII] stage) with the nucleus near the plasma membrane and the presence of a polar body; and (iv) undefined (U) where chromatin could not be visualized or polar body was absent.

Microphotographs were taken for each category using an optical microscope (Leica) with 10× and 20× objectives and an integrated digital camera.

#### Assessment of COC viability

COC from each treatment group were placed in 15 mL conical tubes (SPL-Life Sciences, Korea) containing 1.0 mL of TL-HEPES-PVA medium at 23°C. Tubes were vortexed for 90 s at maximum speed to facilitate cumulus cell removal. Denuded COC were transferred to 5 × 5 cm Petri dishes (BD) and observed under a stereoscopic microscope (Leica). Viability was assessed using trypan blue staining. For each treatment, 10–15 oocytes were placed in a 10 μL drop of trypan blue mixed with 100 μL of TCM-199 medium at 38°C for 5 min. After staining, oocytes were washed for 1 min in TCM-199, mounted on a slide, and covered with a coverslip. Oocytes with blue-stained cytoplasm and zona pellucida were considered non-viable; oocytes without blue staining were considered viable. Microphotographs were obtained using an optical microscope (Leica) with 10× and 20× objectives and a digital camera.

### *In vitro* embryo development

#### IVF

Following *in vitro* maturation, cumulus cells were mechanically removed using a glass Pasteur pipette. Denuded oocytes were washed twice in mTBM fertilization medium (Tris Buffer Modified, In vitro).

For IVF, 30–50 mature oocytes were placed into four-well culture dishes containing 500 μL of mTBM fertilization medium supplemented with 4% fatty acid-free BSA (Fraction V; Sigma-Aldrich) and overlaid with 200 μL of mineral oil (Sigma-Aldrich). Dishes were incubated at 38.5°C under 5% CO^2^ and saturated humidity.

Semen, diluted and refrigerated at 17°C until use, was obtained from a local Livestock Association. Progressive motility of sperm was assessed, and 1.0 mL of diluted semen was centrifuged at 500× *g* for 20 min. The supernatant was immediately discarded, and the cell pellet was reconstituted with 1.0 mL of mTBM fertilization medium (In vitro).

The diluted sperm were incubated at 37°C and transferred to a 1.5 mL microcentrifuge tube. Sperm concentration was estimated by mixing 10 μL of the sperm suspension with HEPES solution (HEPES, In vitro) and adding it to a test tube containing 1990 μL of 0.3% formalin saline solution (1:200 dilution).

After allowing the suspension to settle for 5 min, sperm were counted using a Neubauer chamber (Hausser Scientific, Germany). Sperm concentration was adjusted to 1 × 10^6^ sperm/mL using the fertilization medium.

IVF was carried out by adding 10 μL of sperm suspension to each well containing oocytes. Sperm and oocytes were co-incubated for 7 h at 38.5°C under 5% CO^2^ and saturated humidity.

It is important to note that MLT was not added to the fertilization medium.

#### In vitro embryo development

After fertilization, potential zygotes were washed 3 times with NCSU-23 embryo development medium (North Carolina State University-23, USA) sup-plemented with 4% fatty acid-free BSA (Fraction V; Sigma-Aldrich).

Groups of 20–25 probable zygotes were transferred into four-well dishes containing 500 μL of NCSU-23 medium with BSA, supplemented with varying MLT concentrations: 0.0, 1.0, 3.0, or 5.0 μM.

Thus, four experimental groups were established: CG, consisting of 500 μL of NCSU-23 medium without MLT; EG1 with 500 μL of NCSU-23 medium and 1.0 μM MLT; EG2 with 500 μL of NCSU-23 medium and 3.0 μM MLT; and EG3 with 500 μL of NCSU-23 medium and 5.0 μM MLT.

All culture dishes were overlaid with 200 μL of mineral oil and incubated at 38.5°C in an atmosphere containing 5% CO^2^ and saturated humidity.

After 8 days of culture, embryos were evaluated according to the classification of the International Embryo Technology society:

(i) Cleavage stage: Embryos with 2–8 blastomeres, equal in size, without fragmentation, homogeneous cytoplasm, and an intact zona pellucida with translucent appearance.
(ii) Early morulae: Embryos with 16–32 blastomeres, equal in size, with <10% cytoplasmic fragmentation, intact zona pellucida, and uniform translucent appearance.
(iii) Late morulae: Embryos in which individual blastomeres could not be distinguished due to the onset of compaction, with intact zona pellucida and translucent appearance.
(iv) Early blastocysts: Embryos showing reorganization into two distinct cell populations (embryoblast and trophoblast), with an emerging blastocoel cavity, and maintaining an intact zona pellucida and translucent appearance.

Embryos were evaluated using an inverted microscope (Leica) equipped with a 40× objective, and microphotographs were taken using a USB Digital Camera (OPT-14 mp, Optisum, China).

### Statistical analysis

Percentages for the various categories – mature (MII), maturing (MI), immature (GV), undefined oocytes (U), viable oocytes, and non-viable oocytes – were calculated based on the total number of oocytes used for *in vitro* maturation in each treatment. Each number was divided by the total number of oocytes per treatment and multiplied by 100.

Similarly, the percentages of embryos at different developmental stages (cleavage, morulae, and early blastocyst) were calculated relative to the number of fertilized oocytes in each treatment group.

Data from Experiment I (*in vitro* oocyte maturation) - including the percentages of oocytes at GV, MI, MII, and U stages and oocyte viability - and data from Experiment II (*in vitro* embryo development) - including the percentages of embryos at cleavage, morulae, and early blastocyst stages - were analyzed using analysis of variance (ANOVA) to detect differences between MLT treatments.

*Post hoc* comparisons were performed using Tukey’s test to determine which means significantly differed among treatments. Before conducting ANOVA, percentage data were arcsine transformed to achieve normalization.

Results are presented as mean ± standard error of the mean, and differences were considered statistically significant at p < 0.05. All statistical analyses were performed using the Statistical Package for the Social Sciences (SPSS) software version 15.0 (SPSS Inc., Chicago, IL, USA).

## RESULTS

### Effect of MLT during *in vitro* maturation

**Figure 1:**
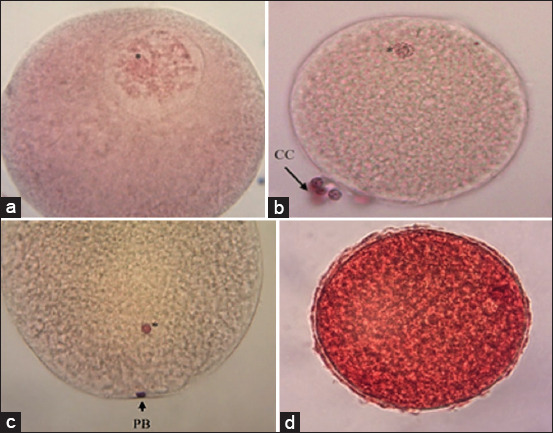
Cumulus-oocyte-complexes (COC) matured *in vitro* for 44 h. Aceto-orcein stain. (a) COC in germinal vesicles. 40× objective. (b) COC immature or Metaphase I. 20× objective. (c) COC mature or Metaphase II, the first polar body is evident. 40× objective. (d) COC undefined. 20× objective. *Nucleus. CC=Cumulus cells, PB=Polar body.

Six replicates were carried out, and a total of 1,142 cumulus-oocyte complexes (COC) were obtained at different stages of maturation: COC that were considered immature or at the GV stage (Figure 1a), COC in the process of maturation or at MI stage (Figure 1b), COC considered mature or at MII stage (Figure 1c), and COC considered undefined (U) (Figure 1d).

Supplementation of the maturation medium with 1.0 μM MLT produced the highest percentage of oocytes reaching Metaphase II (45.9% ± 5.70%); however, no significant differences (p > 0.05) were observed between treatments (Table 1).

**Table 1:** Effects of different concentrations of MLT on COC *in vitro* maturation.

Treatment (µM MLT)	n	Germinal vesicle (GV) (%)	Immature (MI) (%)	Mature (MII) (%)	Undefined (U) (%)
CG (0.0)	269	8.7 ± 1.72	29.8 ± 2.58	34.5 ± 2.46	26.8 ± 5.62
EG1 (1.0)	289	6.2 ± 1.25	33.7 ± 1.98	45.9 ± 5.70	14.0 ± 5.18
EG2 (3.0)	306	8.6 ± 1.22	35.2 ± 1.73	40.7 ± 4.7	15.2 ± 3.10
EG3 (5.0)	278	10.3 ± 2.60	29.5 ± 4.41	43.7 ± 6.46	16.3 ± 6.78

n=number of COC by treatment. Values are expressed as mean ± standard error of the mean. No significant differences (p > 0.05) were observed between treatments. COC=Cumulus-oocyte-complexes, MLT=Melatonin, CG=Control Group, EG=Experimental Group, MI=Metaphase I, MII=Metaphase II

#### Viability of COC exposed to different concentrations of MLT

**Figure 2:**
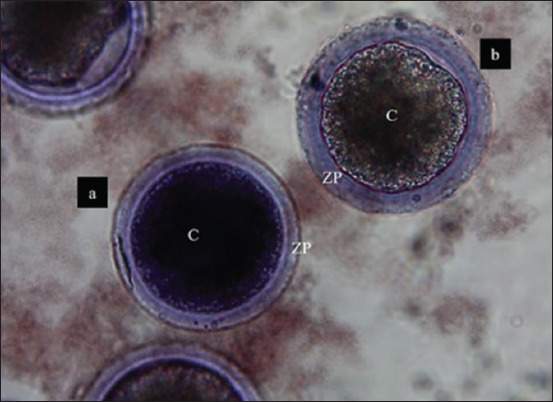
Cumulus-oocyte-complexes matured (COC) *in vitro* for 44 h. Trypan blue stain. (a) Nonviable COC. (b) COC viable. 10× objective. C=Cytoplasm, ZP=Zona pellucida.

Seven replicates were carried out, with a total of 365 COC assessed (Figure 2a and b). Although COC viability ranged between 80.9% and 85.4%, no significant differences (p > 0.05) were found among the MLT treatments (Table 2).

**Table 2:** Effect of different concentrations of MLT on the viability of COC *in vitro*.

Treatment (µM MLT)	n	Viability (%)
CG (0.0)	87	85.4 ± 4.02
EG1 (1.0)	95	80.9 ± 4.94
EG2 (3.0)	87	84.4 ± 4.43
EG3 (5.0)	96	85.0 ± 4.00

n=number of COC by treatment. Values are expressed as mean ± standard error of the mean. No significant differences (p > 0.05) were observed between treatments. COC=Cumulus-oocyte-complexes, MLT=Melatonin, CG=Control Group, EG=Experimental Group

### Effect of MLT during *in vitro* embryo development

**Figure 3:**
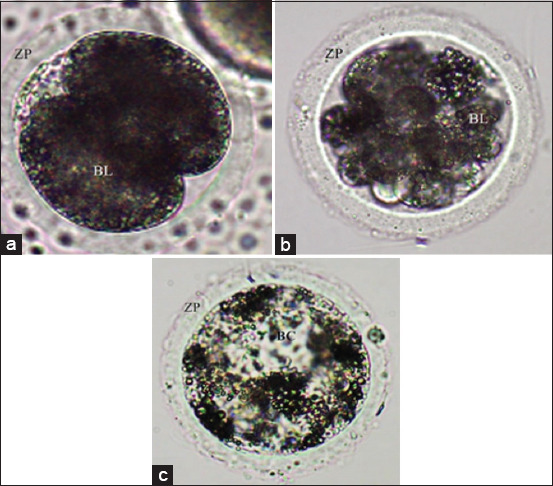
In vitro embryos of pigs exposed to different concentrations of Melatonin. (a) Embryo of two blastomeres (cleavage stage). 20× objective. (b) Early morulae. 20× objective. (c) Early blastocyst. 20× objective. ZP=Zona pellucida, BL=Blastomeres, BC=Blastocoele cavity.

Six replicates were carried out using 1,312 probable zygotes, resulting in 630 embryos obtained at various stages of development: Embryos in the cleavage stage (Figure 3a), early morulae (Figure 3b), late morulae, and early blastocysts (Figure 3c).

Supplementation of the maturation medium with 1.0 μM MLT produced the highest percentage of blastocyst formation (26.9% ± 9.57%); however, no significant differences (p > 0.05) were observed between MLT treatments (Table 3).

**Table 3:** Effects of different concentrations of MLT on *in vitro* embryonic development.

Treatment (µM MLT)	n	Cleavage (%)	Morulae (%)	Early blastocyst (%)
CG (0.0)	336	17.7 ± 4.53	16.6 ± 6.37	14.4 ± 2.33
EG1 (1.0)	340	12.1 ± 4.03	16.7 ± 4.36	26.9 ± 9.57
EG2 (3.0)	316	18.8 ± 4.53	11.9 ± 2.21	21.2 ± 4.43
EG3 (5.0)	320	7.9 ± 3.92	11.5 ± 2.52	15.9 ± 3.11

n=number of potential zygotes according to treatment. Values are expressed as mean ± standard error of the mean. No significant differences (p > 0.05) were observed between treatments. MLT=Melatonin, CG=Control Group, EG=Experimental Group

## DISCUSSION

The pig industry plays a crucial role in livestock farming and veterinary medicine, with productivity management being a key determinant of its success [11]. Furthermore, due to their genetic, anatomical, and physiological similarities, *in vitro*-produced pigs can also serve as suitable organ donors in regenerative medicine and as human disease models.

MLT has been extensively documented as a key regulator of circadian and circannual rhythms and as a potent scavenger of free radicals [7, 12].

Shi *et al*. [6] investigated MLT concentrations in porcine follicles, categorizing them based on diameter: small follicles (3 mm) contained an average of 20 pg/mL, medium follicles (4–8 mm) had 11 pg/mL, and large follicles (>8 mm) had 10 pg/mL. Their findings suggest that lower MLT concentrations may positively influence oocyte maturation. Previous studies by Shi *et al*. [6] and Kang *et al*. [13] reported that supplementing the maturation medium with MLT produced beneficial effects on both nuclear and cytoplasmic maturation of pig oocytes and, consequently, on embryonic development.

Lin *et al*. [14] reviewed optimal MLT con-centrations for porcine culture media, reporting a range of 10¯^11^ M [6], 10 ng/mL [13], 10¯^7^ M [15], and 25 ng/mL [16]. However, the results across studies remain inconclusive and inconsistent. Thus, considering previous information, we decided to test MLT concentrations ranging from 0 to 5 μM during oocyte *in vitro* maturation and embryo *in vitro* development to obtain good-quality embryos.

Zhao *et al*. [17] argued that the development capacity of pig embryos depends mainly on the quality of COC; therefore, a low development rate of COC derived from *in vitro* maturation indicates a low rate of developed blastocysts. In the present study, only high-quality COC (grades 1 and 2) were employed; thus, MLT did not produce positive effects on oocyte *in vitro* maturation and embryo *in vitro* development.

Previous studies by Takada *et al*. [18], Rodrigues-Cunha *et al*. [19], and Méndez *et al*. [20] added smaller amounts of MLT (0.01 μM) to bovine embryos, obtaining no significant results on *in vitro* maturation and cleavage rates. It is worth mentioning that cows and pigs depend on MLT as one of the regulatory hormones of GnRH secretion [9]; furthermore, MLT inhibits specific genes involved in steroidogenesis [21].

In the present work, the maturation medium was not supplemented with steroids or gonadotropins; thus, MLT binds to its receptors in granulosa cells but lacks a companion (which could be hormones). This may preclude MLT effects from taking place. In contrast to this point of view, Rodrigues-Cunha *et al*. [19] argued that serum, follicular fluid, and other hormones present in the oocyte maturation medium could probably interfere with the function of MLT.

Conversely, the MLT concentrations used in this study may have exceeded receptor saturation thresholds, potentially inhibiting inositol triphosphate (IP_3_) and calcium (Ca^2+^) signaling in granulosa cells. Lin *et al*. [14] mentioned that MLT probably plays a key role in cumulus cells rather than in oocytes during maturation.

MLT has been shown to facilitate embryonic development across multiple species, including pigs, cattle, sheep, and mice [6]. The period of supplementation as well as concentration is important factors to obtain positive effects of MLT on pig embryos [16, 22]. The beneficial effects of MLT are related to its ability to improve mitochondrial function and reduce oxidative stress. In addition, it regulates genomic methylation levels that are important during embryonic development [23, 24].

In terms of blastocyst formation rates, our findings surpass those reported by Ishizuka *et al*. [25] in mouse embryos supplemented with 1.0 μM MLT but are lower than those reported by Chaya [26] using 10¯^9^ M MLT in goat embryos. However, in pigs, Currin *et al*. [27] obtained high rates of embryo development to blastocyst (approximately 83%) using a concentration of 100 nM MLT. That concentration is approximately 10 times lower compared to the smallest concentration used in the present work (1.0 μM).

It is possible that the signaling pathway for the synthesis of endogenous MLT in the mitochondria could be altered; it is worth noting that mitochondria are the main organelles responsible for producing adenosine triphosphate and promoting embryonic development. Nevertheless, low MLT concentrations may not improve embryo development and quality, whereas high MLT concentrations may cause embryonic damage due to the production of reactive oxygen species [22].

Considering that the effects of MLT depend on several factors, as previously mentioned, animal species should be considered only one of the factors regulating MLT’s role in both oocyte and embryo *in vitro* culture.

## CONCLUSION

In this study, supplementation with MLT at concentrations of 0.0, 1.0, 3.0, and 5.0 μM during *in vitro* maturation and embryo culture of porcine oocytes was evaluated. Although supplementation with 1.0 μM MLT yielded the highest proportion of oocytes reaching the Metaphase II stage (45.9% ± 5.70%) and the highest blastocyst formation rate (26.9% ± 9.57%), no statistically significant differences were observed across the treatment groups (p > 0.05). Similarly, COC viability remained consistently high (80.9%–85.4%) regardless of MLT concentration, without significant variation.

The practical applicability of this study lies in providing valuable insights into the dose-response relationship of MLT during porcine oocyte maturation and embryo development, emphasizing that simple supplementation with MLT between 0.0 and 5.0 μM does not confer a significant developmental advantage under the tested culture conditions. These findings suggest that MLT alone, in the absence of complementary factors such as gonadotropins or steroid supplementation, may not sufficiently enhance *in vitro* reproductive outcomes in pigs.

A major strength of this study is its rigorous experimental design, which includes the use of high-quality COC (grades 1 and 2), replication of experiments across multiple biological samples, and standardized assessment of maturation, viability, and embryonic development stages. Moreover, the study provides a systematic evaluation of MLT concentrations often cited in the literature but tested here under tightly controlled conditions, allowing for direct comparability.

However, some limitations must be acknowledged. The study was restricted to relatively high concentrations of MLT, potentially exceeding physiological receptor sensitivity. The maturation media were not supplemented with gonadotropins or steroid hormones, factors that could influence MLT receptor activity and downstream effects. In addition, mitochondrial function, oxidative stress markers, and molecular pathways were not evaluated, which limits the mechanistic understanding of the observed outcomes.

Future studies should investigate lower MLT concentrations, particularly in the nanomolar range, to avoid receptor saturation effects. The combinatory effects of MLT with gonadotropins or antioxidants should also be explored. Furthermore, assessing mitochondrial activity, oxidative stress parameters, and epigenetic modifications would provide deeper mechanistic insights into MLT’s role during porcine oocyte maturation and embryo development.

## AUTHORS’ CONTRIBUTIONS

LABA, ALAG, and AAR: Conducted the laboratory experiments and drafted the manuscript. ALAG and AM: Designed and supervised the study, statistical analysis, and revised the manuscript. AM: Project administration. All authors have read and approved the final manuscript.
